# Being a parent of children with attention-deficit hyperactivity disorder

**DOI:** 10.1590/1806-9282.20241335

**Published:** 2025-03-17

**Authors:** Derya Evgin, Nuray Caner, Elif Bilge Alimoğlu, Yağmur Sezer Efe

**Affiliations:** 1 Kayseri University, Faculty of Health Sciences, Child Development – Kayseri, Turkey.; 2 Erciyes University, Faculty of Health Sciences, Department of Pediatric Nursing – Kayseri, Turkey.; 3 Erciyes University, School of Medicine, Department of Child and Adolescent Psychiatry – Kayseri, Turkey.

**Keywords:** Anxiety, Parents, Attention Deficit Disorder with Hyperactivity, Parent-Child Relations

## Abstract

**OBJECTIVE:**

This study was conducted to determine the relationship between stress and anxiety levels of parents of children with attention-deficit hyperactivity disorder and parent–child behaviors.

**METHODS:**

The study was conducted with parents (n=181) of children with attention-deficit hyperactivity disorder who were followed up in the Child Psychiatry Outpatient Clinic of a tertiary hospital in Turkey. Ethics committee approval and institutional permissions were obtained, and data were collected using the Questionnaire Form, Conner's Parent Rating Scale, Alabama Parenting Questionnaire, and State Anxiety Inventory. Descriptive statistics, correlation, and linear regression analyses were used for data analysis.

**RESULTS:**

Of the parents who participated in the study, 86.2% were mothers of children diagnosed with attention-deficit hyperactivity disorder. The mean scores of the Conner's Parent Rating Scale, Alabama positive and negative parental attitudes, and the State Anxiety Inventory were 42.44±17.12, 69.92±7.27, ±37.22±9.23, and 38.29±8.33, respectively. There was a significant positive relationship at a moderate level between the mean scores of the parents’ State Anxiety Inventory, a significant positive relationship at a weak level between the mean scores of the parents’ negative parenting behaviors and Conner's Parent Rating Scale, and a significant negative relationship at a weak level with the mean scores of the parents’ positive parenting behaviors (p<0.05).

**CONCLUSION:**

In this study, it was found that anxiety of parents of children diagnosed with attention-deficit hyperactivity disorder was associated with perceived stress, negative parenting behaviors, and behavioral problems of children.

## INTRODUCTION

Attention-deficit hyperactivity disorder (ADHD), which is characterized by symptoms such as hyperactivity and impulsivity, is a neurodevelopmental disorder frequently encountered in childhood. ADHD is seen in 3–7% of school-age children, and when left untreated, it might lead to various psychological and social problems such as lack of self-confidence, problematic peer relationships, and poor academic achievement^1^. ADHD has profound effects on the psychological health of parents as well as children. Parents of children with ADHD are more likely to experience high levels of anxiety, stress, depression, and other psychological problems^2,3^. High levels of anxiety and depression in parents of children with ADHD can negatively affect family dynamics and parent–child relationships, and this situation worsens the behavioral and emotional problems of children^4^. In particular, children's behaviors that require constant attention and supervision may cause parents to experience high levels of psychosocial problems^5^.

Parents of children with ADHD experience high levels of stress while coping with their children's academic and social challenges, which can predispose them to mood disorders such as depression and anxiety^3^. Alrahili stated that high levels of stress and related mood disorders such as depression and anxiety are more common in parents of children with ADHD^6^. Depressed parents may be intolerant toward their children, which may lead to disruption of family relationships^2^.

Psychological support and interventions for parents of children with ADHD are crucial to the overall health and well-being of both parents and children. Increasing parents’ stress coping skills might play a critical role in reducing psychological problems within the family^6,7^. Parents’ psychological resilience plays an important role in coping with behavioral problems of children with ADHD^8^.

High levels of anxiety in parents of children with ADHD may be caused by both their children's problem behaviors and their own living conditions. Therefore, the psychological health of parents should be taken into account in the ADHD treatment and management process and a holistic approach should be adopted. Increasing parents’ access to psychological support and guidance services can improve the quality of life of both children and parents. In conclusion, examining the relationship between stress and anxiety levels experienced by parents of children diagnosed with ADHD and parent and child behaviors will make important contributions to both academic literature and clinical practice. It is thought that this study will make an important contribution to the literature in this respect.

## METHODS

This cross-sectional and descriptive study was conducted to determine the relationship between stress and anxiety levels of parents of children with ADHD and parent–child behaviors.

The study was conducted with parents (n=181) of children with ADHD who were followed up in the Child Psychiatry Outpatient Clinic of a tertiary hospital located in the Central Anatolia Region of Turkey between 16.01.2023 and 30.06.2023. This study included parents who (1) had a child diagnosed with ADHD, (2) did not have any other mental illness other than ADHD, (3) had no written communication barriers, and (4) volunteered to participate in the study. The exclusion criteria were as follows: (1) parents with schizophrenia, bipolar disorder, or substance abuse and (2) parents currently being treated with psychiatric medication or other psychotherapy. A total of 198 parents applied to the study, of whom 17 were excluded due to missing data. Therefore, a total of 181 parents were included in this study.

The data were collected face-to-face using the State Anxiety Inventory (SAI), Alabama Parenting Questionnaire (APQ), and Conners’ Parent Rating Scale (CPRS).

State Anxiety Inventory (SAI): It was developed by Spielberger et al. to determine the levels of state and trait anxiety separately. The inventory is a self-assessment scale consisting of 20 statements used to determine how the individual feels at a given moment and under certain conditions. A high score indicates a high level of anxiety^9,10^. In this study, Cronbach's alpha for the SAI was calculated as 0.85.

Alabama Parenting Questionnaire (APQ): The scale examines parental behaviors in five different domains: "Parental Involvement (PI), Positive Parenting (PP), Poor Monitoring (PM), Inconsistent discipline (ID), and Corporal Punishment (CP)." While the PI and PP subscales measure positive parenting behaviors, PM, ID, and CP subscales measure negative parental behaviors^11^. In this study, the Cronbach's alpha for the APQ was calculated as 0.70–0.72.

Conners’ Parent Rating Scale (CPRS): The scale was developed to measure behavior problems in children. The scale consists of 48 items in total and includes 5 subscales: "Behavior Problems, Hyperactivity, Learning Problems, Anxiety, and Psychosomatics." A high score indicates that the child has the specified problem behaviors^12^. In this study, Cronbach's alpha for the CPRS was calculated as 0.86.

The study was conducted between January and June 2023 with parents (n=181) of children diagnosed with ADHD admitted to the Child Psychiatry Outpatient Clinic of a tertiary hospital. Data were collected using the Questionnaire Form, State Anxiety Inventory (SAI), Alabama Parenting Questionnaire (APQ), and Conner's Parent Rating Scale (CPRS). The average time for filling out the questionnaire is 15–20 min.

The data were analyzed using SPSS 25.0 (IBM Corp., Armonk, New York, USA). Data are shown as number (n), percentages (%), mean (X), and standard deviations (SD). The Shapiro-Wilk test, QQ plots, and histograms were performed to test the normality of the data. The Kruskal-Wallis H test and Spearman correlation analysis were used to evaluate the data. In line with the established models, multiple regression was applied to investigate the effect of independent variables on dependent variables. The power of the study was calculated using the "G. Power-3.1.9.2" program. The Cronbach α value was calculated for the scales used in the study. The results were evaluated at 95% confidence interval, and the significance level was accepted as p<0.05.

This research complies with the provisions of the Declaration of Helsinki (as revised in Brazil, 2013). Ethics committee approval (02/12/2022-81) and institutional permissions were obtained to conduct the study. Written and verbal consent was obtained from all participants, and their anonymity was preserved.

## RESULTS

Of the parents who participated in the study, 86.2% were female and 50.8% had secondary education. The mean age of the parents was 38.55±6.09 years, and the mean scores of the CPRS, Alabama positive and negative parental attitudes, and the SAI were 42.44±17.12, 69.92±7.27, ±37.22±9.23, and 38.29±8.33, respectively. The mean ages of children diagnosed with ADHD at the time of ADHD diagnosis, current age, and duration of ADHD treatment were 7.60±2.69, 10.52±3.29, and 2.76±3.91 years, respectively.

There was a significant positive relationship at a moderate level between the mean scores of the parents’ SAI, a significant positive relationship at a weak level between the mean scores of the parents’ negative parenting behaviors and CPRS, and a significant negative relationship at a weak level with the mean scores of the parents’ positive parenting behaviors (p<0.05) (Table 1).

**Table 1:** The relationship between Conner's Parent Rating Scale, Alabama Parenting Questionnaire, and State Anxiety Inventory mean scores of parents.

	1	2	3
1. CPRS	1		
2. Total Alabama positive behavior	-0.124	1	
3. Total Alabama negative behavior	0.330*	-0.223*	1
4. SAI	0.248*	-0.290*	0.297*

Spearman's correlation analysis.

* p<0.01.

CPRS: Conners’ Parent Rating Scale; SAI: State Anxiety Inventory.

Parents’ gender, education level, and the status of thinking that the discipline methods they applied were effective were not related to CPRS, SAI, and Alabama negative parenting attitudes. However, it was found that the mean scores of the Alabama positive parenting attitude scale were higher in parents with higher levels of education and who thought that the discipline methods they applied were effective (Table 2).

**Table 2:** The relationship between parents’ descriptive characteristics and mean scores on the Conner's Parent Rating Scale, Alabama Parenting Questionnaire, and State Anxiety Inventory.

Descriptive Characteristics		CPRS		Alabama positive behavior		Alabama negative behavior		SAI	
		Mean±SD	Min–max	Mean±SD	Min–max	Mean±SD	Min–max	Mean±SD	Min–max
Gender of parent									
	Female	42.85±16.67	10.0–86.0	66.17±7.15	44.0–80.0	36.90±9.20	17.0–72.0	38.20±8.36	20.0–61.0
	Male	39.88±19.93	13.0–97.0	64.40±7.97	49.0–80.0	39.20±9.32	26.0–57.0	39.91±8.32	22.0–54.0
		t=0.317	p=0.485	t=0.788	p=0.304	t=0.332	p=0.261	t=0.037	p=0.698
Education level									
	Primary school	37.69±16.3	10.0–64.0	65.34±7.01	44.0–74.0	35.53±6.94	25.0–56.0	37.73±7.88	20.0–57.0
	Secondary school	44.36±16.81	13.0–82.0	64.55±8.01	47.0–80.0	38.29±9.09	23.0–71.0	38.57±7.99	20.0–58.0
	Higher education	41.58±17.70	13.0–97.0	68.17±5.59	58.0–80.0	36.34±10.14	17.0–72.0	38.12±9.09	22.0–61
		F=1.673	p=0.191	F=4.938	**p=0.038**	F=1.339	p=0.265	F=0.122	p=0.885
Thinking that disciplinary methods are effective									
	Yes	34.11±15.28	10.0–64.0	65.05±8.17^ab^	50.0–76.0	36.17±8.04	17.0–49.0	37.05±6.44	29.0–46.0
	No	44.47±15.86	16.0–86.0	63.69±7.93^b^	44.0–80.0	38.56±8.14	24.0–61.0	39.25±7.85	25.0–61.0
	Sometimes	42.27±17.91	13.0–97.0	67.73±6.11^a^	52.0–80.0	36.09±9.54	20.0–72.0	37.72±8.99	20.0–58.0
		F=2.057	p=0.131	F=6.593	**p=0.002**	F=1.573	p=0.210	F=0.807	p=0.448

Statistically significant values are indicated in bold. CPRS: Conners’ Parent Rating Scale; SAI: State Anxiety Inventory; SD: SD: standard deviation.

The superscripts "a" and "b" indicate that the Alabama positive parenting scores of mothers who did not find the disciplining methods effective and those who sometimes found them effective were statistically significantly different.

Linear regression analysis was performed to determine the factors associated with APQ scores in parents of children diagnosed with ADHD, and three different models were created to determine the associated factors. In Model 1, the characteristics of the parents and their children were examined and it was found that the dependent variables explained 8.2% of the mean Alabama negative parenting scores (p=0.021). In Model 2, in addition to the characteristics of the parents and their children, the total number of disciplinary methods used by the parents was added and it was found that the dependent variables explained 13% of the mean Alabama negative parenting scores (p=0.001). Model 3 was found to be the best model to explain negative parenting attitudes in parents of children diagnosed with ADHD. In the third model, the characteristics of the parents and their children, the total disciplinary methods applied by the parents, and the mean scores of CPRS and SAI explained 29% of the mean scores of Alabama negative parental attitudes of the parents (p=0.001) (Table 3).

**Table 3:** Factors associated with parents’ Alabama negative parental attitudes.

Determinants		Alabama negative parental attitudes						
		β	SE	β	p	95%CI	Adj. R^2^	Model p
Model 1								
	Constant	32.37	5.066		0.001	22.354–42.394	0.082	0.021
	Education level	-0.351	1.143	-0.027	0.759	-2.611 to 1.909		
	Gender of parent^a^	-3.193	2.602	-0.106	0.222	-8.339 to 1.953		
	Age at diagnosis of ADHD	0.856	0.284	0.291	0.003	0.295–1.417		
	Duration of treatment	-0.474	0.306	-0.149	0.123	-1.078 to 0.131		
Model 2								
	Constant	29.969	5.000		0.001	20.079–39.858	0.137	0.001
	Education level	-0.699	1.119	-0.053	0.533	-2.911 to 1.514		
	Gender of parent^a^	-2.890	2.535	-0.096	0.256	-7.903 to 2.124		
	Age at diagnosis of ADHD	0.975	0.279	0.332	0.001	0.423–1.527		
	Duration of treatment	-0.615	0.301	-0.194	0.043	-1.211 to −0.019		
	Total disciplining methods	1.906	0.656	0.239	0.004	0.609–3.204		
Model 3								
	Constant	16.837	5.457		0.001	6.042–27.633	0.293	0.001
	Education level	-1.063	1.025	-0.080	0.302	-3.091 to 0.965		
	Gender of parent^a^	-3.421	2.321	-0.114	0.143	-8.014 to 1.171		
	Age at diagnosis of ADHD	0.993	0.258	0.338	0.001	0.483–1.503		
	Duration of treatment	-0.593	0.276	-0.187	0.034	-1.139 to −0.047		
	Total disciplining methods	1.473	0.605	0.185	0.016	0.277–2.670		
	CPRS	0.160	0.041	0.307	0.001	0.079–0.241		
	SAI	0.206	0.087	0.184	0.019	0.035–0.378		
Durbin Watson: 2.026; VIF: 1.044–1428								

B: unstandardized Beta; SE: standard error; β: standardized beta β; R^2^: correlation coefficient (explained variance ratio); p: level of significance; CI: confidence interval; ADHD: attention-deficit hyperactivity disorder; CPRS: Conners’ Parent Rating Scale; SAI: State Anxiety Inventory; VIF: variance inflation factor.

a While coding, female was coded as 1 and male was coded as 0.

## DISCUSSION

The mean scores obtained from the scales used in the study provide important clues in understanding the psychological and parenting status of parents of children diagnosed with ADHD. High levels of anxiety, depression, and stress are common in parents of children with ADHD^4^. In the present study, the mean score of the parents on the SAI was 38.29±8.33, indicating that the parents experienced high levels of anxiety. This result reveals that parents of children diagnosed with ADHD are prone to mood disorders and their psychological health can be seriously affected. Alrahili^6^ and Erden and Uzun^13^ showed that parents of children diagnosed with ADHD have high levels of depression, anxiety, and stress, which negatively affect their overall quality of life. It was reported that parents of children with ADHD had impaired family harmony and negatively affected parent–child interactions due to high levels of stress and anxiety^2^.

The mean score of positive parental attitudes obtained from the Alabama Parenting Questionnaire was 69.92±7.27, indicating a high level of positive parenting behaviors. A high score for positive behaviors in the scale indicates that parents display a supportive, loving, and constructive attitude toward their children. This can have a positive impact on children's emotional and social development. This may have a positive effect on children's emotional and social development. The mean score of negative parental attitudes was 37.22±9.23, indicating the presence of negative behaviors at a certain level. However, this score may also indicate a lower level of negative behaviors. This suggests that parents may occasionally exhibit negative behaviors in moments of discipline or stress, but overall positive attitudes are more dominant. That is, parents exhibit both positive and negative behaviors.

Parents of children with ADHD may experience significant levels of anxiety and stress due to their children's special needs and behavioral problems. This may cause parents to have negative parenting attitudes. In this study, linear regression analysis was conducted to determine the factors associated with the Alabama negative parenting attitudes scores of the parents of children diagnosed with ADHD, and three different models were created to determine the associated factors. In Model 1, the characteristics of parents and children were examined and it was found that the dependent variables explained 8.2% of the mean Alabama negative parenting attitudes scores (p=0.021). This result suggests that demographic and personal characteristics of parents and children have a limited effect on negative parenting attitudes. In the literature, it is stated that the effect of such characteristics on negative parenting attitudes is generally limited^14^. Parents’ demographic characteristics had a limited effect on their success in managing their children's ADHD symptoms^5^. In Model 2, in addition to the characteristics of the parents and their children, the total number of disciplinary methods used by the parents was added and it was found that the dependent variables explained 13% of the mean Alabama negative parenting scores (p=0.001). This suggests that the discipline methods applied by parents have a more significant effect on negative parenting attitudes. Discipline methods determine how parents respond to and manage their children's behavioral problems. Effective discipline methods can promote more positive parenting attitudes by reducing parents’ stress and anxiety levels^15^. It was found that the model that best explained the negative parenting attitudes in parents of children diagnosed with ADHD was Model 3. In Model 3, the characteristics of the parents and their children, the total disciplinary methods used by the parents, and the mean scores of the CPRS and the SAI explained 29% of the mean scores of the parents’ Alabama negative parental attitudes (p=0.001). This result suggests that a more comprehensive approach is more effective in understanding and explaining parents’ negative attitudes. Parents’ psychological resilience and general psychological health may have a significant effect on their parenting attitudes^8^.

## CONCLUSION

In the study, parents’ gender, educational level, and finding effective discipline methods were not directly related to the negative parenting attitudes of the CPRS, the SAI, and the APQ, but parents with higher educational level and finding discipline methods effective had higher positive parenting attitudes. These results reveal important points to consider in the design of support programs for parents of children with ADHD.
